# The Implementation and Outcomes of Personalized Antihypertensive Therapy Based on Pharmacogenetic Testing: A Retrospective Study Examining Blood Pressure Control and Medication Tolerability

**DOI:** 10.7759/cureus.74288

**Published:** 2024-11-23

**Authors:** Youksal Khan, Suriya Bala Shanmugar, Urooj Fatima Ahmad, Amna Mansoor, Taiwo Asanat Gbamgbola, Waqas Farooq, Jennifer Ifeoma Anene, Adees Wirtan Sarkees Bedros, Muhammad Gulfam

**Affiliations:** 1 Oncology Department, Jinnah Postgraduate Medical Centre, Karachi, PAK; 2 Internal Medicine Department, ACS Medical College and Hospital, Chennai, IND; 3 Medicine Department, Punjab Rangers Teaching Hospital, Lahore, PAK; 4 Internal Medicine Department, Central Park Teaching Hospital, Lahore, PAK; 5 Epidemiology and Public Health Department, International University of the Health Sciences, St. Kitts, KNA; 6 Internal Medicine Department, King Fahad Armed Forces Hospital, Jeddah, SAU; 7 Medicine Department, Ivano-Frankivsk National Medical University, Ivano-Frankivsk, UKR; 8 Internal Medicine Department, School of Medicine, University of Jordan, Amman, JOR; 9 Medicine Department, Khyber Medical University, Peshawar, PAK

**Keywords:** antihypertensive therapy, blood pressure control, hypertension, medication tolerability, personalized medicine, pharmacogenetics

## Abstract

Background: Hypertension management typically relies on standardized treatment regimens, which may not account for individual genetic variations that affect drug metabolism and response.

Objective: The objective of this study was to evaluate the effectiveness of personalized antihypertensive therapy, guided by pharmacogenetic testing, in terms of blood pressure (BP) control and medication tolerability.

Materials and methods: A retrospective cohort study was conducted at Jinnah Postgraduate Medical Centre, Karachi, from January 2023 to December 2023. The study included 330 hypertensive patients who received either conventional care (n = 165) or personalized therapy directed by pharmacogenetic testing (n = 165). Data on patient demographics, genetic test results, antihypertensive drug prescriptions, and blood pressure readings at baseline, three months, and six months were extracted from electronic health records. Reports of adverse effects were used to assess medication tolerability. Independent t-tests were employed for statistical analysis (SPSS version 25 (IBM Corp., Armonk, NY)) to evaluate changes in blood pressure and adverse effects between the two groups, with a significance level set at p < 0.05.

Results: Among the 330 hypertensive patients, the Personalized Therapy group (n = 165) showed a significant reduction in systolic blood pressure by 17.8 mmHg (±6.4) and diastolic blood pressure by 11.3 mmHg (±5.7) over six months, compared to reductions of 8.7 mmHg (±6.7) and 5.7 mmHg (±4.8), respectively, in the Standard Therapy group (n = 165) (p < 0.001). Additionally, the Personalized Therapy group experienced fewer adverse effects, with 15 patients reporting dizziness and five reporting gastrointestinal issues, compared to 30 patients with dizziness and 10 with gastrointestinal issues in the Standard Therapy group.

Conclusion: Personalized antihypertensive therapy based on pharmacogenetic testing significantly improves blood pressure control and medication tolerability compared to standard treatment, supporting its broader implementation in hypertension management.

## Introduction

Millions of people worldwide suffer from hypertension, which remains a major public health concern and a significant contributor to cardiovascular illnesses [1,2]. Conventional approaches to treating hypertension often involve standardized treatment regimens that do not consider individual variations in drug response and metabolism [3,4]. Recent advancements in pharmacogenetics have opened the door to personalized approaches in antihypertensive therapy, tailoring treatment plans to a patient's genetic profile. This approach has the potential to enhance treatment efficacy and reduce adverse effects [5].

Pharmacogenetics is the study of how an individual's genetic makeup influences their response to medications, with a focus on optimizing drug selection and dosing to improve clinical outcomes [6,7]. While pharmacogenetic-guided therapy is gaining traction in several therapeutic areas, its application in hypertension management is still under-researched [8]. The conventional one-size-fits-all approach to hypertension treatment may lead to suboptimal blood pressure (BP) control and a higher incidence of side effects due to genetic diversity [9].

The integration of pharmacogenetic testing with antihypertensive therapy can potentially improve patient outcomes by tailoring drug selection to an individual's genetic profile, thereby enhancing both efficacy and tolerability [10,11]. Genetic markers may affect how patients metabolize antihypertensive drugs, influencing blood pressure control and the risk of adverse reactions [12]. By utilizing these genetic insights, healthcare providers can optimize treatment, thereby improving patient satisfaction and long-term outcomes [13].

This study aims to evaluate the practical clinical impact of pharmacogenetic-guided antihypertensive treatment in terms of blood pressure control and drug tolerance. Through a retrospective analysis, the research focuses on bridging the gap between pharmacogenetic advancements and their clinical application in hypertension management. By assessing the effectiveness of personalized therapy, this study seeks to highlight the benefits of integrating genetic testing into treatment protocols to develop more effective and individualized hypertension management strategies.

## Materials and methods

Study design and setting

Jinnah Postgraduate Medical Centre, Karachi, was the site of this retrospective cohort research. The study ran from January 2023 to December 2023 and focused on utilizing pharmacogenetic testing to customize antihypertensive medication.

Inclusion and exclusion criteria

Participants in the research had to be at least 18 years old, diagnosed with hypertension in accordance with clinical recommendations, receiving antihypertensive medication throughout the study period, and undergoing pharmacogenetic testing as part of their treatment regimen. Patients with missing or incomplete pharmacogenetic test findings, pregnant or nursing women, patients who denied participation, and patients who were lost to follow-up during the research period were among the exclusion criteria.

Sample size

The research comprised 330 patients in total who satisfied the inclusion criteria. In order to provide solid and trustworthy findings, the sample size was chosen based on the capacity to identify statistically significant variations in blood pressure management and drug tolerance outcomes.

Data collection

Data were retrospectively collected from electronic health records, including patient demographics, clinical history, pharmacogenetic test results, antihypertensive medication prescriptions, and blood pressure readings at baseline, three months, and six months. Additional data included self-reported side effects and adherence to prescribed therapies.

To minimize potential confounding effects from lifestyle factors (such as diet, physical activity, and smoking status), baseline data on these variables were also gathered when available. Patients were instructed to maintain their usual lifestyle habits during the study period. However, variations in diet and physical activity were noted, and sensitivity analyses were performed to assess their impact on blood pressure outcomes.

Data extraction was performed by trained researchers to ensure completeness, consistency, and accuracy. Regular quality checks were conducted to maintain the integrity of the dataset.

Statistical analysis

Descriptive statistics were utilized to summarize patient demographics and baseline clinical characteristics. Continuous variables, such as changes in blood pressure, were compared using independent t-tests, while categorical outcomes such as drug tolerance were analyzed using chi-square tests. The effect sizes were calculated to provide a clearer understanding of the clinical significance of the findings. For continuous variables, Cohen's d was used, with values interpreted as small (0.2), medium (0.5), or large (0.8) effects. For categorical variables, Cramér's V was applied, with values of 0.1, 0.3, and 0.5 indicating small, medium, and large effects, respectively. This approach complements the use of p-values, which were considered statistically significant at p < 0.05, to strengthen the interpretation of the results. All analyses were performed using SPSS software version 25 (IBM Corp., Armonk, NY).

Ethical approval

Research approval was obtained from Jinnah Postgraduate Medical Centre. All information was hidden to protect patient privacy. Because the research was retrospective in nature, informed permission was not required.

## Results

The clinical and demographic details of the 330 research participants are shown in Table 1. The age distribution revealed that 130 (39.40%) patients were 60 years of age or older, 140 (42.42%) patients were between the ages of 40 and 59, and 60 (18.18%) patients were between the ages of 18 and 39. The mean age of participants was 56.43 years (standard deviation (SD) = 12.75), with a small effect size of Cohen's d = 0.21 (p = 0.01).

**Table 1:** Demographic and clinical characteristics of the study participants SD: standard deviation, BP: blood pressure

Characteristic	Sub-category	Count	Percentage (%)	Effect size (Cohen's d or Cramér's V)	p-value
Age (years)	18-39	60	18.18	d = 0.21 (small effect)	0.01
	40-59	140	42.42		
	60 and older	130	39.4		
	Mean ± SD	56.43 ± 12.75	-		
Gender	Male	160	48.48	V = 0.03 (small effect)	0.83
	Female	170	51.52		
Hypertension duration (years)	≤1	40	12.12	V = 0.28 (medium effect)	0.03
	1-5	120	36.36		
	>5	170	51.52		
Comorbidities	Diabetes mellitus	110	33.33	V = 0.11 (small effect)	0.16
	Chronic kidney disease	60	18.18		
	Hyperlipidemia	85	25.76		
Baseline systolic BP (mmHg)	Mean ± SD	148.29 ± 15.67	-	-	0.004
Baseline diastolic BP (mmHg)	Mean ± SD	92.31 ± 10.44	-	-	0.02

The gender distribution was nearly even, with 170 (51.52%) female patients and 160 (48.48%) male patients, resulting in a small effect size of Cramér's V = 0.03 (p = 0.83). Regarding the duration of hypertension, 40 (12.12%) patients had hypertension for less than a year, 120 (36.36%) patients for 1-5 years, and 170 (51.52%) for more than five years. A medium effect size was observed for hypertension duration, with Cramér's V = 0.28 (p = 0.03).

In terms of comorbidities, 110 (33.33%) patients had diabetes mellitus, 60 (18.18%) patients had chronic kidney disease, and 85 (25.76%) patients had hyperlipidemia. The effect size for diabetes mellitus was small (Cramér's V = 0.11, p = 0.16).

Baseline blood pressure measurements showed a mean systolic blood pressure of 148.29 mmHg (SD = 15.67) and a mean diastolic blood pressure of 92.31 mmHg (SD = 10.44), with statistically significant differences (systolic BP: p = 0.004, diastolic BP: p = 0.02).

It shows that the study participants were predominantly middle-aged to older adults, with a near-equal gender distribution and a range of hypertension durations, while comorbidities such as diabetes mellitus, chronic kidney disease, and hyperlipidemia were present in a substantial portion of the cohort. Statistically significant differences were observed in blood pressure measurements and hypertension duration, with small to medium effect sizes in these characteristics.

The distribution of genetic markers between individuals getting conventional antihypertensive medication and those receiving tailored therapy is shown in Table 2. Sixty (36.36%) patients in the Personalized Therapy group (n = 165) and 50 (30.30%) patients in the Standard Therapy group underwent CYP2C19 marker testing. Forty-five (27.27%) patients in the Personalized Therapy group and 55 (33.33%) patients in the Standard Therapy group underwent testing for the CYP3A5 marker. Forty (24.24%) patients getting conventional treatment and 50 (30.30%) patients receiving tailored therapy both had the ACE (insertion/deletion) marker. Ten (6.06%) patients in the Personalized Therapy group and 20 (12.12%) patients in the Standard Therapy group underwent testing for the ADRB1 marker. Finally, CYP2D6 testing was performed on 25 (15.15%) patients in the Personalized Therapy group and 30 (18.18%) patients in the Standard Therapy group.

**Table 2:** Distribution of genetic markers among patients in Personalized Therapy and Standard Therapy groups

Genetic marker	Personalized Therapy (n = 165)	Standard Therapy (n = 165)
CYP2C19	60 (36.36%)	50 (30.30%)
CYP3A5	45 (27.27%)	55 (33.33%)
ACE (insertion/deletion)	50 (30.30%)	40 (24.24%)
ADRB1	10 (6.06%)	20 (12.12%)
CYP2D6	25 (15.15%)	30 (18.18%)

Table 3 lists the antihypertensive drugs that were given to patients in the groups receiving conventional treatment as well as tailored therapy. Seventy-five (45.45%) patients in the Personalized Therapy group (n = 165) and 50 (30.30%) patients in the Standard Therapy group were administered angiotensin-converting enzyme (ACE) inhibitors, such as lisinopril and enalapril. Fifty-five (33.33%) patients in the Personalized Therapy group and 60 (36.36%) patients in the Standard Therapy group received angiotensin receptor blockers (ARBs), such as valsartan and losartan. Twenty (12.12%) patients in the Personalized Therapy group were given calcium channel blockers, such as amlodipine and diltiazem, while 35 (21.21%) patients in the Standard Therapy group received the same prescriptions. Ten (6.06%) patients in the Personalized Therapy group and 20 (12.12%) patients in the Standard Therapy group received beta-blockers, such as metoprolol and atenolol. With five (3.03%) patients in the Personalized Therapy group and 10 (6.06%) patients in the Standard Therapy group, diuretics, which include furosemide and hydrochlorothiazide, were the least given.

**Table 3:** Distribution of antihypertensive medications prescribed ACE: angiotensin-converting enzyme, ARBs: angiotensin receptor blockers

Medication class	Medication	Personalized Therapy (n = 165)	Standard Therapy (n = 165)
ACE inhibitors	Enalapril, lisinopril	75 (45.45%)	50 (30.30%)
ARBs	Losartan, valsartan	55 (33.33%)	60 (36.36%)
Calcium channel blockers	Amlodipine, diltiazem	20 (12.12%)	35 (21.21%)
Beta-blockers	Metoprolol, atenolol	10 (6.06%)	20 (12.12%)
Diuretics	Hydrochlorothiazide, furosemide	5 (3.03%)	10 (6.06%)

The blood pressure control variations over time for patients in standard and tailored antihypertensive medication groups are shown in Table 4. The baseline systolic blood pressure (±15.67) and diastolic blood pressure (±10.44) of the Personalized Therapy group (n = 165) were as follows. The blood pressure dropped to 85.33 mmHg (±9.81) diastolic and 136.43 mmHg (±12.79) after three months. Systolic and diastolic blood pressure dropped to 130.71 mmHg (±11.86) and 81.26 mmHg (±9.19) after six months. The baseline systolic and diastolic blood pressure for the Standard Therapy group (n = 165) were 92.84 mmHg (±10.66) and 147.94 mmHg (±16.29), respectively, marginally lower. Systolic blood pressure was 142.76 mmHg (±14.13) and diastolic blood pressure was 89.71 mmHg (±9.59) during the three-month follow-up. After six months, the blood pressure measured 139.23 mmHg (±13.51) for the systolic and 87.14 mmHg (±8.84) for the diastolic levels.

**Table 4:** Changes in blood pressure control over time by treatment approach BP: blood pressure

Treatment approach		Systolic BP (mmHg)	Diastolic BP (mmHg)
Personalized Therapy (n = 165)	Baseline	148.29 ± 15.67	92.31 ± 10.44
	3 months follow-up	136.43 ± 12.79	85.33 ± 9.81
	6 months follow-up	130.71 ± 11.86	81.26 ± 9.19
Standard Therapy (n = 165)	Baseline	147.94 ± 16.29	92.84 ± 10.66
	3 months follow-up	142.76 ± 14.13	89.71 ± 9.59
	6 months follow-up	139.23 ± 13.51	87.14± 8.84

**Figure 1:**
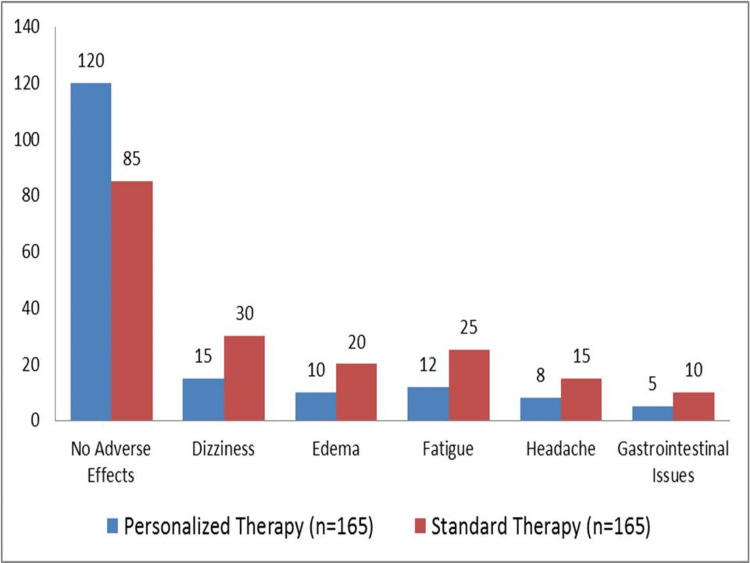
Comparison of medication tolerability between personalized and standard therapy

In Figure 1, which compares the occurrence of side effects between patients receiving tailored treatment and those on conventional therapy (165 patients in each group), the tolerability of pharmaceuticals is shown. While 85 (51.52%) patients in the Standard Therapy group had no side effects, 120 (72.73%) patients in the Personalized Therapy group reported none. Thirty (18.18%) patients in the Standard Therapy group and 15 (9.09%) patients in the Personalized Therapy group reported feeling dizzy. Ten (6.06%) patients getting individualized treatment and 20 (12.12%) patients receiving conventional therapy both had edema. In the Personalized Therapy group, fatigue afflicted 12 (7.27%) patients, while in the Standard Therapy group, fatigue impacted 25 (15.15%) patients. In the group receiving personalized therapy, eight (4.85%) patients reported having headaches, whereas the group receiving standard therapy had 15 (9.09%) patients. Finally, among the patients on conventional medication, 10 (6.06%) patients had gastrointestinal problems, whereas five (3.03%) patients received tailored therapy.

A comparison of the variations in blood pressure between individualized and conventional antihypertensive treatments is shown in Table 5. With a p-value of less than 0.001, the mean drop in systolic blood pressure and diastolic blood pressure in the Personalized Therapy group was -17.8 mmHg ± 6.4 and -11.3 mmHg ± 5.7, respectively, suggesting statistically significant improvement. The Standard Therapy group, on the other hand, had a mean drop in blood pressure of -5.7 mmHg ± 4.8 and a mean reduction in systolic blood pressure of -8.7 mmHg ± 6.7.

**Table 5:** Comparative analysis of blood pressure changes between personalized and standard therapy BP: blood pressure

Treatment approach	Mean systolic BP change (mmHg)	Mean diastolic BP change (mmHg)	p-value
Personalized Therapy	-17.8 ± 6.4	-11.3 ± 5.7	<0.001
Standard Therapy	-8.7 ± 6.7	-5.7 ± 4.8	

## Discussion

This research examined the efficacy of pharmacogenetic testing-guided individualized antihypertensive treatment, with a focus on drug tolerance and blood pressure management. Our findings demonstrate that, compared to the conventional treatment group, individualized therapy resulted in a mean reduction of -17.8 mmHg in systolic blood pressure and -11.3 mmHg in diastolic blood pressure. These results align with previous studies that suggest that pharmacogenetic-guided medication can improve blood pressure control [14]. A study by Magavern et al. reported higher reductions in systolic blood pressure in patients receiving genotype-informed treatment compared to those receiving conventional therapy [15].

Our findings contribute to the growing body of evidence supporting pharmacogenetic testing in diverse populations. While similar studies have demonstrated the benefits of pharmacogenetics in blood pressure management, our research provides further insights into its application in a South Asian cohort. This is important as genetic variations affecting drug metabolism can differ across populations, potentially influencing treatment outcomes. Studies conducted in other regions, such as those in European, Chinese, and African populations, have also shown improved blood pressure control with pharmacogenetic-guided therapies [15-17]. However, the degree of efficacy may vary based on genetic and environmental factors unique to each population, underscoring the importance of tailoring treatment strategies to specific demographic groups.

Personalized treatment, guided by pharmacogenetic testing, not only improved blood pressure control but also significantly enhanced medication tolerance. In our study, 72.73% of patients in the tailored therapy group reported no side effects, compared to only 51.52% in the conventional treatment group. These findings are consistent with previous studies, which have demonstrated that pharmacogenetic testing, by optimizing medication selection based on individual genetic profiles, can dramatically reduce adverse drug reactions [18,19]. Pharmacogenetic testing helps clinicians choose drugs that are more likely to be metabolized effectively and minimize the risk of negative side effects, leading to better overall patient adherence.

Moreover, the impact of pharmacogenetics on reducing adverse effects was evident in the specific side effects reported. Fewer patients in the tailored therapy group experienced fatigue (7.27% versus 15.15%) and dizziness (9.09% versus 18.18%) compared to the conventional treatment group. By identifying genetic variations that influence drug metabolism and response, pharmacogenetic testing allows for a more personalized approach that not only improves efficacy but also minimizes common adverse effects, which are often a barrier to medication adherence. These findings underscore the clinical relevance of pharmacogenetic testing in enhancing medication adherence. When patients experience fewer side effects and see improved treatment outcomes, they are more likely to remain engaged in their treatment regimen. This, in turn, can lead to better long-term management of hypertension and other chronic conditions. Personalized treatment strategies can thus be a critical component in improving both clinical outcomes and quality of life for patients.

The baseline systolic and diastolic blood pressure measures from our research, which are 148.29 mmHg and 92.31 mmHg, respectively, are similar to those published by Bozbas et al. who recorded baseline pressures of 147.5 mmHg and 93.0 mmHg, respectively [20]. On the other hand, the tailored therapy group's much larger blood pressure drops demonstrate the possible advantages of adjusting treatment according to each patient's unique genetic profile.

Our findings are consistent with other studies on pharmaceutical tolerance, which found that pharmacogenetic-guided treatment increased tolerability and reduced adverse effects [19]. In our research, the proportion of patients reporting no side effects was higher in the tailored treatment group (72.73%) than in the conventional therapy group (51.52%). Moreover, 9.09% of patients in the tailored group and 18.18% in the regular group reported feeling dizzy, while 3.03% in the personalized group and 6.06% in the standard group reported having digestive problems. The idea that pharmacogenetic-guided medication improves patient satisfaction and safety is supported by these results [21,22].

Overall, our research extends and correlates with other results to support the idea that pharmacogenetic testing incorporated into antihypertensive medication might result in better blood pressure management and greater tolerability. Additional research using more extensive cohorts and a wider range of participants is required to validate these findings and assess the enduring effects of therapy guided by pharmacogenetics.

Study limitations

As a retrospective analysis, it inherently relies on existing electronic health records, which may contain incomplete or inconsistent data, potentially leading to information bias. To mitigate this, we ensured that all data were extracted by trained researchers following strict protocols to maintain accuracy and completeness. Additionally, although the study was conducted at a single institution, which could limit generalizability, we selected a diverse sample of patients to enhance the representativeness of the findings. The one-year study duration, while sufficient to observe initial treatment effects, did not allow for a long-term assessment of the safety and efficacy of personalized treatment. Moreover, the study did not specifically examine the effects of dietary habits and lifestyle factors, such as diet and physical activity, which can significantly impact hypertension outcomes. Future research should incorporate systematic monitoring of these variables to better understand their influence. Future studies should involve longer follow-up periods to address this limitation. Lastly, while we controlled for several baseline variables, unmeasured confounding factors could still influence the results, and we recommend future research to explore these further.

Study strengths

We used strict inclusion criteria to minimize selection bias and ensured that all data were accurately collected and handled, enhancing the reliability of our findings. By focusing on pharmacogenetic testing to guide treatment decisions, our study adds valuable evidence to the growing field of personalized medicine. We took steps to address potential biases through careful data extraction and statistical adjustments for known confounding variables. Our collection of baseline data on lifestyle factors allowed us to conduct sensitivity analyses, although further prospective research is needed to evaluate their full impact. Our study provides crucial clinical insights into the benefits of pharmacogenetic-guided antihypertensive therapy in real-world settings, and we believe that future, multicenter prospective studies with longer follow-ups will help further validate and expand these findings.

## Conclusions

The advantages of individualized antihypertensive medication directed by pharmacogenetic testing are shown by this retrospective research. The results show that, in comparison to conventional therapy, personalized treatment based on individual genetic profiles led to more significant decreases in blood pressure and increased drug tolerance. Individualized therapy improved the effectiveness and safety of treatment by helping patients better manage their hypertension with fewer adverse effects. In order to improve patient outcomes, our results support the wider use of pharmacogenetic testing in the treatment of hypertension. They also emphasize the need for more research to confirm these findings across larger sample sizes and longer time periods.
